# Hybrid Communication Architecture and Flexible Multi-Parameter Sensing Modules for Mine Rescue: Design and Preliminary Validation

**DOI:** 10.3390/s26092629

**Published:** 2026-04-24

**Authors:** Shengyuan Wang, Peng Chen, Shiyang Peng, Jiahao Liu

**Affiliations:** 1 School of Emergency Management and Safety Engineering, China University of Mining and Technology-Beijing, Beijing 100083, China; 2 School of Mine Safety, University of Emergency Management, Beijing (East Yanjiao), Beijing 065201, China

**Keywords:** mine rescue, emergency communications, flexible sensing, vital signs

## Abstract

Mine rescue operations are frequently conducted in hazardous underground environments characterized by damaged infrastructure, unstable communications, heat stress, and hypoxia risk, all of which threaten the safety of rescue personnel. To address these challenges, this study proposes a prototype-oriented mine-rescue monitoring framework that combines a Wi-Fi/optical-fiber communication architecture with flexible wearable sensing modules for physiological monitoring. The communication design employs Wi-Fi for local wireless data aggregation and optical fiber for reliable long-distance backhaul to the surface command side. For wearable monitoring, two flexible sensing modules were developed: a temperature sensor based on a polyaniline/graphene–polyvinyl butyral composite film and a PPG-oriented flexible optoelectronic module based on an ITO/Ag/ITO multilayer transparent electrode structure. Experimental results show that the temperature sensor exhibits a clear temperature-dependent resistance response within the tested range, while the optoelectronic module demonstrates low sheet resistance and acceptable electrical continuity under repeated bending. These results provide preliminary support for combining hybrid underground communication architecture with flexible wearable sensing components in mine-rescue scenarios. However, the present work remains at the stage of architecture design and component-level validation, and full end-to-end system verification under simulated or field rescue conditions will be the focus of future studies.

## 1. Introduction

Mine accidents continue to pose severe threats to human safety and economic stability worldwide, despite significant advancements in mining safety technologies. In China, the implementation of the Three-Year Action Plan for Special Rectification of Production Safety has notably reduced accident frequency, yet underground emergencies—particularly in coal mines—remain challenging due to complex geological conditions and potential secondary disasters [1,2] (e.g., gas explosions, roof collapses). Emergency rescue operations under such conditions are often hampered by communication failures and a lack of real-time data, leading to prolonged rescue cycles, inefficient decision-making, and elevated risks for the rescue team [3,4]. Existing mine rescue systems rely heavily on either wired (e.g., optical fiber) or wireless (e.g., RF, acoustic) communication [5]. Wired networks offer stability but are vulnerable to infrastructure damage in disasters, while wireless solutions face signal attenuation, interference, and limited bandwidth in underground environments. Moreover, most commercial sensors focus on environmental gas monitoring but neglect real-time physiological monitoring of rescuers, whose vital signs (e.g., body temperature, blood oxygen, and heart rate) are critical for preventing casualties during high-intensity operations. Flexible wearable sensors, though emerging in healthcare, are rarely tailored for mine rescue scenarios. Recent studies have begun to extend wearable physiological monitoring from conventional healthcare settings to high-risk occupational and disaster-related environments. For example, recent reviews have highlighted the growing role of wearable sensors in monitoring industrial workers under physically demanding and thermally stressful conditions [6]. In parallel, wearable medical sensor systems have been reported for vital-sign monitoring in disaster scenarios [7]. Moreover, real-time wearable technologies for occupational heat-stress assessment have further demonstrated the importance of continuous monitoring of parameters such as body temperature, cardiovascular response, and related physiological strain under extreme environmental exposure [8]. However, these recent advances have been developed mainly for general industrial, healthcare, or disaster-response contexts, rather than for underground mine rescue scenarios characterized by communication obstruction, confined space, high humidity, dust exposure, and intense body motion [9,10,11,12,13]. Consequently, a clear gap remains in the development of a mine-rescue-oriented framework that combines underground communication support with wearable physiological sensing modules capable of maintaining structural adaptability and stable operation under harsh rescue conditions.

To address these gaps, this study proposes a hybrid mine-rescue monitoring framework that combines underground communication architecture with flexible wearable sensing modules. The main contributions of this work are summarized as follows:

- A hybrid Wi-Fi/optical-fiber communication architecture is proposed for mine-rescue scenarios, combining the deployment flexibility of local wireless transmission with the reliability of a wired backbone for long-distance backhaul.
- Two flexible sensing modules are developed for physiological monitoring, including a temperature sensor based on a polyaniline/graphene–polyvinyl butyral composite film and a PPG-oriented flexible optoelectronic module based on an ITO/Ag/ITO multilayer transparent electrode structure, with emphasis on wearability and structural adaptability under bending deformation.
- A unified design framework is established to connect underground communication architecture with wearable physiological sensing components, thereby providing the technical basis for future synchronized monitoring of environmental and physiological information in mine rescue operations.

The remainder of this paper is organized as follows. Section 2 describes the hybrid communication architecture and the main equipment configuration required for mine-rescue deployment. Section 3 explains the rationale for selecting vital-sign parameters for physiological monitoring and discusses their clinical and operational relevance in mine-rescue scenarios. Section 4 presents the fabrication, characterization, and preliminary electrical/mechanical validation of the developed flexible sensing modules. Section 5 discusses the limitations of the current work and outlines future research directions. Finally, the main findings of this study are summarized. Overall, this work aims to provide a prototype-oriented technical basis for intelligent and human-centered mine-rescue monitoring.

## 2. Hybrid Communication Network

### 2.1. Selection of Wired Communication Networks

Given the distinctive characteristics of underground mining environments and the stringent requirements for communication transmission, this study adopts a wired–wireless hybrid networking architecture for mine rescue communication systems, which simultaneously prioritizes both usability and reliability. More precisely, the proposed approach employs wired communication for medium-to-long-distance transmission and wireless communication for short-distance links. This integrated strategy maintains the inherent flexibility of wireless networking while effectively mitigating the limitations associated with purely wireless systems, such as poor reliability and unstable data transmission. Wired communication technologies are generally recognized for their stability, reliability, and security. A summary of commonly deployed underground wired communication technologies and their respective characteristics is presented in Table 1.

Coaxial cable demonstrates satisfactory performance in conventional video transmission; however, its physical characteristics constrain its applicability in confined underground spaces, and the associated cabling complexity undermines its suitability for mine rescue communication systems. Twisted pair cabling, despite its lower cost, is inadequate for long-distance or high-bandwidth scenarios due to inherent signal attenuation and electromagnetic susceptibility. In contrast, optical fiber communication offers three critical advantages—high bandwidth enabling real-time high-definition video backhaul, robust immunity to electromagnetic interference in complex mining environments, and low attenuation ensuring reliable signal integrity over extended distances—thereby fulfilling the functional requirements of long-haul transmission in rescue operations. Although the deployment of optical fiber entails higher installation costs, this limitation can be effectively alleviated through engineering strategies such as the utilization of pre-terminated cables, which reduce on-site termination complexity and deployment time.

### 2.2. Selection of Wireless Transmission Technologies

Common wireless transmission technologies used in underground mines include WiFi, RFID, Bluetooth, and ZigBee. Their characteristics and parameters are compared in Table 2, respectively.

In the context of mine rescue operations, Wi-Fi technology is considered a preferred solution for wireless communication, owing to its substantial advantages in addressing critical operational requirements, including high-bandwidth support for real-time video and data transmission, extended coverage in complex underground environments, rapid and flexible deployment, and robust resistance to electromagnetic interference. Specifically, Wi-Fi enables transmission rates of up to 54 Mbps, significantly surpassing the capabilities of Bluetooth and ZigBee, thereby facilitating seamless high-definition video streaming. Its coverage range extends up to 300 m in open areas, rendering it suitable for both confined tunnels and expansive underground zones. Moreover, Wi-Fi supports both peer-to-peer ad hoc and infrastructure modes, allowing for dynamic adaptation to variable rescue scenarios without the need for physical cabling. The incorporation of the Carrier Sense Multiple Access with Collision Avoidance (CSMA/CA) protocol and Orthogonal Frequency Division Multiplexing (OFDM) further enhances its resilience against electromagnetic interference generated by mining equipment. Although the initial deployment cost of Wi-Fi systems may be relatively high, the elimination of cabling requirements and the ease of implementation contribute to their comprehensive superiority over alternatives such as RFID, Bluetooth, and ZigBee. These attributes render Wi-Fi particularly well-aligned with the core demands of mine rescue operations.

### 2.3. Construction of a Mixed Network Communication System for Mine Rescue

**Figure 1:**
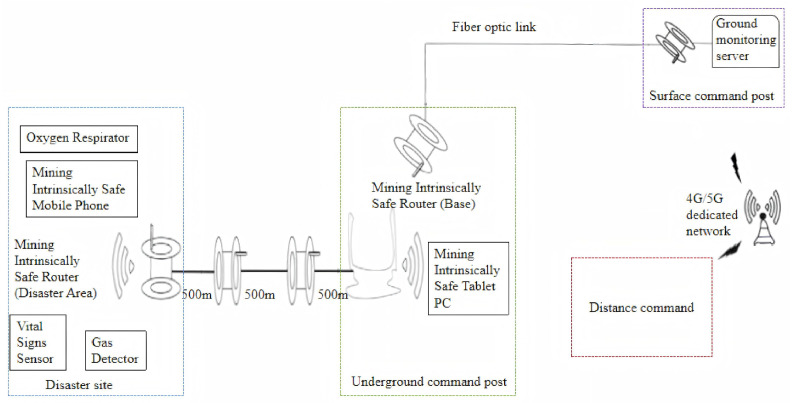
Schematic diagram of rescue communication system.

As depicted in Figure 1, the mine rescue communication system integrates intrinsically safe mobile phones, tablets, routers, gas meters, and vital sign sensors to establish a Wi-Fi network at the rescue site. This network enables voice/video communication and sensor deployment, with wireless modules transmitting real-time environmental and physiological data from the disaster area to the underground base’s tablet for on-site assessment. Fiber optic links relay these data to the surface command post, ensuring uninterrupted information flow that enhances situational awareness and supports informed tactical decisions. The recommended equipment list is shown in Table 3.

## 3. Selection of Vital Sign Parameters

### 3.1. Body Temperature Parameters

In mine disaster rescue scenarios, personnel are required to operate for prolonged periods in enclosed environments characterized by high temperature, high humidity, and restricted ventilation. These conditions involve intense physical exertion while wearing closed-circuit protective equipment, resulting in the superposition of metabolic heat production and environmental heat load, which markedly increases the risk of heat stress and heat-related diseases (e.g., heat cramps, heat exhaustion, and heat stroke). Studies have demonstrated that the underground hot–humid environment not only reduces operational efficiency but also impairs the ability to perform tasks safely [20]. Authoritative occupational health guidelines explicitly recommend the establishment of medical surveillance and on-site physiological monitoring systems to enable early identification of adverse heat-related outcomes [21].

From a physiological perspective, the central objective of occupational heat-exposure management is to maintain core body temperature below 38 °C, a commonly accepted upper limit of tolerable heat strain and a benchmark used in occupational exposure standards [22]. Field measurements from comparable high-heat-load occupations (e.g., firefighting) indicate that, under conditions of elevated ambient temperature and strenuous activity, workers’ core temperature may rise from approximately 37.5 °C to 38.4 °C (and may approach 39.6 °C post-exposure), accompanied by a significant increase in heart rate. These findings suggest that sustained heat exposure imposes a substantial physiological burden, with potential consequences for cognition and work performance [23]. The specific reference indicators of body temperature are as shown in Table 4.

Accordingly, in the high-risk heat-exposure context of mine rescue operations, the use of core body temperature as a key monitoring indicator—combined with dynamic surveillance of physiological parameters such as heart rate—provides not only a scientific basis for evaluating individual heat tolerance and establishing work–rest regimens, but also an essential medical strategy for preventing heat injury and ensuring the operational safety and decision-making capacity of rescue personnel.

### 3.2. Heart Rate Parameters

In mine disaster rescue operations, personnel are exposed to hot, humid, and poorly ventilated underground environments while performing physically demanding tasks in encapsulating personal protective equipment (PPE). The combination of environmental heat load and metabolic heat production creates a typical scenario of uncompensable heat stress. Field simulations have shown that rescuers operate at a mean of 78.6% of their age-predicted maximal heart rate, with peaks reaching 94.5%, while underground temperatures frequently exceed 40 °C with relative humidity around 60%, substantially increasing the risk of elevation of the core temperature [24]. Similarly, studies of live-fire suppression reported mean maximal heart rates of 177±23 beats·min^−1^ within 30 min of activity, with on-scene cardiovascular strain often exceeding that observed during routine exercise testing, indicating that laboratory evaluations may underestimate real emergency workloads [25].

Protective equipment further restricts heat dissipation and promotes heat storage, thereby contributing to the development of heat-related illness [26]. In heat-exposed workers, heart rate is closely associated with metabolic demand and thermophysiological strain and can be continuously monitored using wearable technologies [27]. Field-based investigations have also demonstrated that elevations relative to resting heart rate can effectively predict the risk of heat-related illness [28], and similar exceedances of recommended heart rate limits have been observed in other high-temperature occupations, suggesting a consistent physiological mechanism across job sectors [29]. The criteria for heart rate are shown in Table 5.

For the references used in this table, the following equations were applied [24,28]: The maximum heart rate is calculated as:

(1) $$HR_{\max}=220-age$$

The relative heart rate is defined as:

(2) $$\%HR_{\max}=\frac{HR_{\mathrm{work}}}{HR_{\max}}\times 100\%$$

The relative increase from resting heart rate is given by:

(3) $$\Delta HR_{\mathrm{rest}}=\frac{HR_{\mathrm{peak}}-HR_{\mathrm{rest}}}{HR_{\mathrm{rest}}}\times 100\%$$

Accordingly, continuous heart rate monitoring in mine rescue settings provides an objective means to detect early heat maladaptation and to guide work–rest rotation and cooling strategies under extreme operational conditions.

### 3.3. Blood Oxygen and Oxygen Parameters

In mine disaster rescue environments, roof collapse, combustion-induced oxygen consumption, ventilation disruption, and the accumulation of hazardous gases can rapidly reduce the partial pressure of oxygen within the workspace, thereby creating a risk of insidious hypoxia. Continuous monitoring of peripheral oxygen saturation (SpO_2_) in rescue personnel is therefore a critical component of occupational medical support systems.

Field epidemiological investigations have shown that during underground mining operations, approximately 35% of workers exhibit hypoxemia with SpO_2_ values below 93%, and about 10% experience declines to below 80%. Such levels may impair cognitive function and markedly increase the risk of asphyxiation, indicating that hypoxic exposure in underground environments can directly induce physiological oxygen deficiency even in individuals without pre-existing cardiopulmonary disease [30].

Similarly, studies have demonstrated that in poorly ventilated mining areas, low-oxygen conditions significantly affect rescuers’ work performance and physiological workload, whereas supplemental oxygen can improve blood oxygenation and attenuate stress responses such as elevated heart rate [31]. Occupational safety standards further specify that workplaces with oxygen concentrations below 19.5% are defined as oxygen-deficient atmospheres requiring respiratory protection and continuous monitoring, as tissue oxygen delivery below this threshold cannot sustain normal physiological function [32].

When oxygen concentration falls below 16%, decrements in attention, impaired coordination, and rapid fatigue may occur, while further reductions can lead to errors in judgment or even loss of consciousness. NIOSH guidelines also emphasize that oxygen level assessment and continuous monitoring must be conducted in environments where oxygen deficiency may exist to prevent unrecognized hazardous exposure [33].

The corresponding blood oxygen and environmental oxygen indicators used in this study are summarized as Table 6.

Based on this evidence, oxygen saturation, as a direct physiological indicator reflecting the status of oxygen transport and utilization in the body, enables real-time warning along the pathological cascade from environmental hypoxia to tissue hypoxia and subsequent functional impairment. Owing to its noninvasive, continuous, and field-deployable characteristics, Based on this evidence, oxygen saturation (SpO_2_), it can facilitate early identification of occult hypoxia, guide work–rest rotation and oxygen supplementation strategies, and, when integrated with heart rate and core temperature, form a multiparameter monitoring framework to reduce secondary accidents caused by asphyxia or impaired decision-making capacity.

## 4. Flexible Sensors for Smart Wearable Vital Signs Monitoring

### 4.1. Design of a Flexible Body Temperature Sensor

#### 4.1.1. Materials for Temperature Sensor Fabrication

In this study, considering the requirements for flexibility and wearable applications, a flexible body temperature sensor was designed based on a polyaniline/graphene (GPANI)–polyvinyl butyral (PVB) composite film integrated on an indium tin oxide (ITO)–polyethylene terephthalate (PET) substrate.

GPANI, serving as the conductive filler, was uniformly dispersed within the polymer matrix, while polyvinyl butyral (PVB) acted as the film-forming binder. Anhydrous ethanol was used as the dispersing solvent to ensure homogeneous mixing. The flexible substrate consisted of ITO-patterned PET films (thickness: 0.13 mm; size: 75 mm × 75 mm). The substrate surface was patterned with ten parallel strip-shaped ITO electrodes, each with a width of 4 mm and an inter-electrode spacing of 1 mm.

#### 4.1.2. Preparation of GPANI–PVB Composite Film

**Figure 2:**
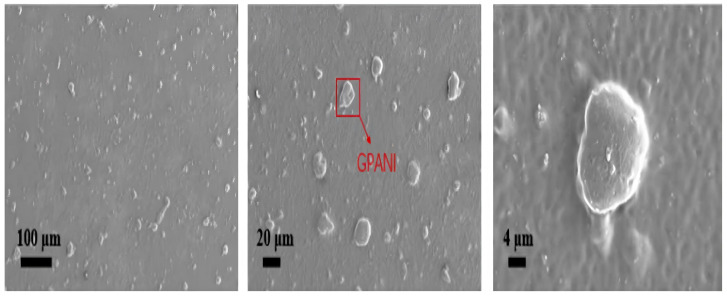
Scanning electron microscope (sem) image [34].

Reference [34], the preparation of GPANI-PVB composite membrane was carried out as follows: GPANI, PVB, and anhydrous ethanol were mixed at a weight ratio of 0.1:5:100. The mixture was first subjected to magnetic stirring and subsequently to ultrasonic dispersion to obtain a homogeneous GPANI–PVB precursor solution. Ethanol was selected as the solvent due to its ability to promote stable dispersion of GPANI particles. The GPANI content was controlled at 0.1 wt% to ensure sufficient electrical conductivity while maintaining optical transparency, whereas the PVB concentration was fixed at 5 wt% to provide adequate mechanical stability and favorable film-forming properties. As shown in Figure 2, scanning electron microscopy (SEM) characterization reveals that GPANI particles are uniformly dispersed within the PVB matrix.

The resulting precursor solution was uniformly deposited onto the ITO–PET substrate to form a wet film, followed by thermal annealing at 80 °C for 15 min to remove residual solvent and enhance the interfacial interaction between GPANI and PVB. After annealing, a GPANI–PVB composite film with a thickness of approximately 2.7 μm was obtained.

In the GPANI–PVB composite system, the GPANI/PVB ratio synergistically determines the final material performance. GPANI serves as the functional conductive filler and mainly regulates the electrical and optical properties. When GPANI is uniformly dispersed in the PVB matrix at a low loading level as microparticles (e.g., 10–20 μm), effective conductive pathways can be established, and the GPANI density primarily determines the baseline conductivity of the composite film [35]. Notably, in this type of sensor, the pressure and temperature sensitivities are governed mainly by the microscopic contact mechanism of GPANI particles rather than by the GPANI concentration itself, making it possible to maintain high sensitivity while reducing power consumption through low-loading design [34,35]. In addition, low GPANI loading minimizes light absorption and scattering, thereby preserving high optical transparency, with visible transmittance exceeding 96% in the pressure-sensing film [35]. By contrast, PVB acts as the continuous flexible matrix, and its concentration and molecular weight are critical to the mechanical stability and film-forming behavior. High-molecular-weight PVB improves tensile strength and toughness through chain entanglement and intermolecular interactions, but also increases solution viscosity, requiring a balance between mechanical robustness, film quality, and processability [36]. Moreover, good film-forming behavior generally requires sufficient PVB content, and a PVB fraction above 30 wt% was reported to be favorable for stable film formation [37].

#### 4.1.3. Device Fabrication

A second ITO-PET substrate was placed on top of the GPANI–PVB composite film, with the ITO electrodes on the top layer aligned perpendicularly to those on the bottom substrate. This configuration forms a cross-electrode structure, generating multiple temperature-sensing units at the intersections of the upper and lower electrodes. The cross-array architecture enables the formation of an array of sensing pixels and improves the spatial resolution of temperature detection. Finally, the top and bottom substrates were fixed and encapsulated along the edges using frame tape to ensure mechanical stability of the device. Through the above fabrication process, a flexible temperature sensor based on the GPANI–PVB composite film was successfully prepared.

### 4.2. Temperature Sensor Stability Test

GPANI particles are embedded within and partially penetrate the transparent polyvinyl butyral (PVB) film, forming a vertically conductive pathway across the sensing layer. At relatively low pressures, slight mechanical deformation of the GPANI particles occurs, leading to resistance variations that are simultaneously influenced by both applied pressure and temperature [38]. This dual dependence enables the sensor to exhibit coupled pressure–temperature sensing capability. When the applied pressure exceeds approximately 30,000 Pa, further deformation of the GPANI particles becomes negligible, and the pressure-induced resistance variation diminishes. Under this condition, the electrical resistance is predominantly governed by temperature, allowing the sensor to operate effectively as a temperature sensor.

#### 4.2.1. Experimental Necessity: Temperature and Pressure Characteristics

Temperature and bending characterizations are essential for evaluating the sensing performance and mechanical reliability of flexible temperature sensors. As temperature is the primary stimulus detected by the device, systematic thermal testing is required to verify the sensor’s sensitivity, linearity, and operational stability over the target temperature range.

Furthermore, flexible sensors designed for wearable electronics or electronic skin applications are frequently subjected to repeated mechanical deformation during practical use. Therefore, bending tests are necessary to assess the structural integrity and functional stability of the sensing layer under mechanical strain. Such characterization also enables evaluation of whether mechanical deformation induces signal interference or degrades the temperature-sensing performance of the device.

#### 4.2.2. Mechanism: Electrical Conduction Behavior of GPANI Composites

The temperature-sensing mechanism of the device originates from the temperature-dependent electrical conductivity of the GPANI composite network embedded within the transparent polyvinyl butyral (PVB) film. The electrical transport behavior of GPANI is governed by the competition between thermally activated carrier excitation and lattice scattering. At relatively low temperatures, carrier excitation dominates, resulting in an increase in conductivity with increasing temperature. As the temperature further increases, lattice scattering becomes increasingly significant, which limits carrier mobility and suppresses further conductivity enhancement. This thermally dependent transport behavior forms the fundamental basis for resistance-based temperature sensing in GPANI composite materials.

#### 4.2.3. Performance Evaluation Metrics

To quantitatively evaluate the sensing performance, several key parameters are typically characterized through temperature and mechanical tests [39]. Temperature measurements are used to determine the temperature coefficient of resistance (TCR), which reflects the sensitivity of the sensor to temperature variations within the target operating range (e.g., 25–80 °C).

The linearity between resistance change and temperature is commonly assessed using the coefficient of determination (e.g., $R^{2}>0.99$). In addition, cyclic temperature and bending tests are performed to evaluate the stability and durability of the device. Metrics such as resistance variation after repeated cycling (e.g., <10% change after thousands of cycles) and signal fluctuation are used to assess long-term reliability under both thermal and mechanical stimuli.

#### 4.2.4. Test Method and Results (Temperature Sensor)

For temperature-dependent resistance performance testing, different pressures were applied to the sensor using a pressure gauge, while the ambient temperature was controlled using a temperature controller. Simultaneously, under a relative humidity of 75% and within the temperature range of 20–80 °C, the resistance variations of the sensor were measured using a digital multimeter.

**Figure 3:**
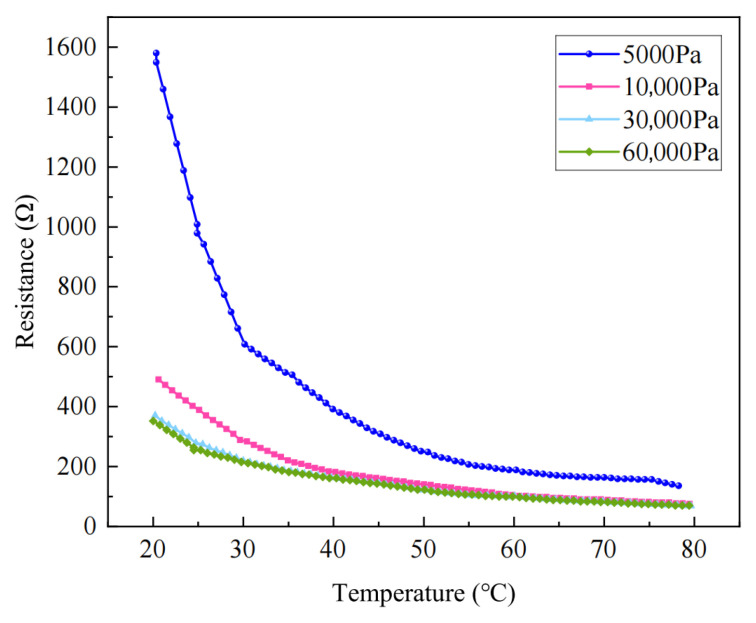
Various temperatures—resistance change graph.

The temperature–resistance characteristics under different conditions are presented in Figure 3. It can be observed that the material exhibits a negative temperature coefficient (NTC) behavior, where the resistance decreases with increasing temperature. In addition, at a given temperature, higher applied pressure results in lower resistance values.

From the perspective of pressure dependence, under low pressure (e.g., 5000 Pa), the resistance–temperature curve is steeper, indicating a significant decrease in resistance with increasing temperature. In contrast, under higher pressures (e.g., 30 and 60,000 Pa), the curve becomes flatter, and the resistance shows a smaller variation with temperature. Specifically, over the range of 20–80 °C, the resistance decreases from approximately 350 Ω to 50 Ω, suggesting reduced temperature sensitivity under high-pressure conditions.

The normalized resistance can be calculated using the following equation [34,35]. The normalized resistance is defined as follows:

(4) $$R_{n}=\frac{R(T)}{R_{0}}$$

where R(T) is the resistance at temperature *T* (in Ω), $R_{n}$ is the normalized resistance, and $R_{0}$ is the resistance at the reference temperature, where $R_{0}$ is taken as the resistance at T=25 °C.

The temperature sensitivity S(T) is calculated as follows:

(5) $$S\left(T\right)=\frac{d(R_{n})}{d(T)}$$

where S(T) is the temperature sensitivity, with the unit of °C^−1^.

Since the experimental data are discrete points, S(T) is calculated using the central difference method:

(6) $$S\left(T_{i}\right)=\frac{{\left(R_{n}\right)}_{i+1}-{\left(R_{n}\right)}_{i-1}}{T_{i+1}-T_{i-1}}$$

where $T_{i}$ is the current temperature point, $T_{i+1}$ is the next temperature point, and $T_{i-1}$ is the previous temperature point; i=1,2,3,….

**Figure 4:**
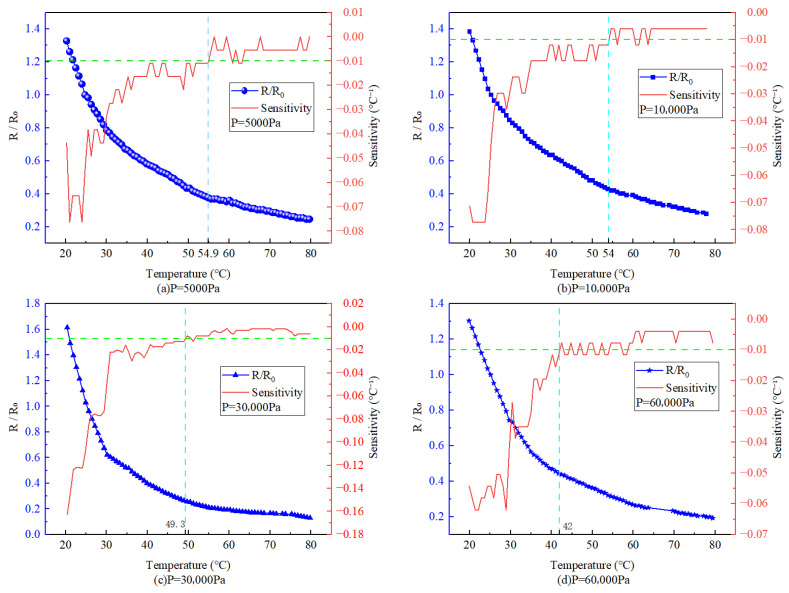
Various temperatures—normalized resistance change graph.

The normalized resistance results are presented in Figure 4. It is evident that under all four pressure conditions, $R_{n}$ decreases exponentially with increasing temperature. A green reference line corresponding to $S\left(T\right)=0.01\;{}^{\circ}C^{-1}$ is included in the figure for comparison. Under low-pressure conditions (P=5000Pa), the temperature sensor reaches this threshold only when the temperature rises to 54.9 °C. Even under high-pressure conditions (P=60,000Pa), the threshold is reached at 42 °C. Referring to the previous discussion on temperature performance indicators, the temperature sensitivity of the sensor satisfies the requirements for practical applications.

Based on the temperature test results, it is evident that the polyaniline/graphene (GPANI) composite integrates the electrical conductivity of polyaniline (PANI) with the high electron mobility and thermal stability of graphene nanoplatelets (GNPs). Its electrical conductivity exhibits strong temperature dependence, which can be attributed to the competition between carrier excitation and lattice scattering mechanisms.

At low temperatures, carrier excitation dominates, resulting in an increase in conductivity with temperature. Beyond a critical temperature, lattice scattering becomes predominant, thereby suppressing further enhancement of conductivity.

Furthermore, embedding GPANI particles within a transparent polyvinyl butyral (PVB) matrix enables pressure-tunable characteristics. Under low pressure (<30,000 Pa), slight deformation of the particles causes the resistance to be influenced by both pressure and temperature, enabling dual-parameter sensing. Under high pressure (>30,000 Pa), particle deformation saturates, and the contact state stabilizes, such that the resistance is primarily governed by temperature, allowing accurate temperature measurement.

### 4.3. Design of a PPG-Oriented Flexible Optoelectronic Module

#### 4.3.1. Principle and Rationale for Selecting PPG-Based Physiological Monitoring

Electrocardiography (ECG) and photoplethysmography (PPG) are two commonly used techniques for monitoring heart rate and blood oxygen saturation. ECG measures the electrical activity of the heart through electrodes attached to the body and provides accurate physiological information. However, the requirement for multiple electrodes and relatively complex instrumentation limits its application in wearable devices.

Photoplethysmography (PPG) is a non-invasive optical technique used to detect blood volume changes in peripheral vessels. In wearable systems, a light-emitting diode (LED) illuminates the skin, and a photodetector measures the reflected or transmitted light intensity. Because tissues such as muscle and bone exhibit relatively constant optical absorption, the periodic variation in detected light intensity mainly results from pulsatile arterial blood flow, enabling the PPG signal to represent the pulse waveform.

#### 4.3.2. Fabrication of the Flexible Optoelectronic Module for PPG-Oriented Sensing

**Figure 5:**
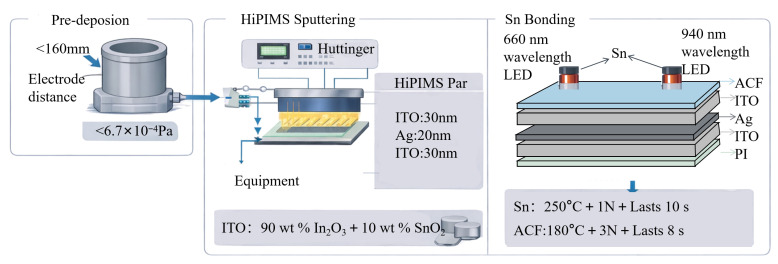
Schematic diagram of the fabrication process for flexible optoelectronic module.

In this study, a flexible PPG-oriented optoelectronic module was fabricated by integrating a transparent conductive multilayer (TCML) film with dual-wavelength LEDs at 660 nm and 940 nm.The specific fabrication process is illustrated in Figure 5. First, a 50 μm-thick colorless polyimide (PI) film was used as the flexible substrate. An ITO–Ag–ITO transparent conductive multilayer film was deposited by high-power impulse magnetron sputtering (HiPIMS), providing a transparent electrode with high optical transmittance, low sheet resistance, and good mechanical flexibility.The deposition process parameters are as shown in Table 7.

Second, an Sn interconnection layer was bonded to the vertical infrared LED at 250 °C under a pressure of 1 N for 10 s. Third, the LED chip was integrated and electrically connected to the TCML/PI substrate using an anisotropic conductive film (ACF). The LED parameters in Table 8 (among these, the recommended drive mode, recommended peak current window, and recommended timing sequence are shared between the two channels) are recommended design targets based on the literature, since the present study focuses mainly on the ITO–Ag–ITO transparent conductive multilayer and its flexible module integration rather than the full device-level validation of the dual-wavelength LED emission unit. Finally, a transparent adhesive was injected between the TCML/PI layer and the flexible circuit substrate to enhance the structural stability of the device while maintaining optical transmittance.

Based on the above processes, a flexible PPG-oriented optoelectronic module employing dual-wavelength LEDs at 660 nm and 940 nm as the light sources was successfully fabricated, providing a hardware platform for the acquisition of physiological photoelectric signals.

#### 4.3.3. Conductivity Test

The sheet resistance and resistivity data obtained from Hall effect measurements for the individual ITO layer deposited by HiPIMS, as well as for various multilayer structures, are shown in Table 9.

When testing a single 200 nm-thick ITO film, it is difficult to directly integrate it onto a 50 μm-thick PI substrate due to limitations in mechanical compatibility and process stability. The experimental results, as summarized in Table 9, indicate that introducing an Ag interlayer between two ITO layers significantly reduces both the sheet resistance and the resistivity of the composite film.

Specifically, when the Ag thickness increases from 10 nm to 20 nm, the sheet resistance decreases from 23.7 Ω/sq to 6.5 Ω/sq, while the resistivity decreases from $9.3\times 10^{-4}$ Ω·cm to $2.5\times 10^{-5}$ Ω·cm. Similarly, when the Ag layer is sandwiched between two ITO layers of 30 nm thickness on each side, a comparable reduction in resistance is observed, a comparable reduction in resistance is observed.

These results highlight that optimizing the thickness of each layer in the multilayer structure, particularly the Ag interlayer, is an effective strategy for reducing overall resistance and enhancing electrical conductivity. In addition, multilayer architectures can alleviate internal stress and improve interfacial adhesion and surface flatness on the PI substrate.

Therefore, Ag interlayers are incorporated into successive ITO layers to simultaneously achieve low thickness and low resistivity. The optical transmittance of the 30 nm ITO/20 nm Ag/30 nm ITO structure exceeds 86% at 660 nm and 88% at 940 nm (typical PPG wavelengths), ensuring more than 95% relative signal-to-noise ratio (SNR) compared with bare ITO.

#### 4.3.4. Fabrication of the Flexible Optoelectronic Sensor

**Figure 6:**
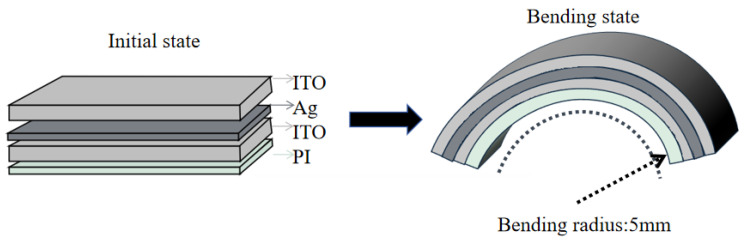
Schematic diagram of the bending method.

**Figure 7:**
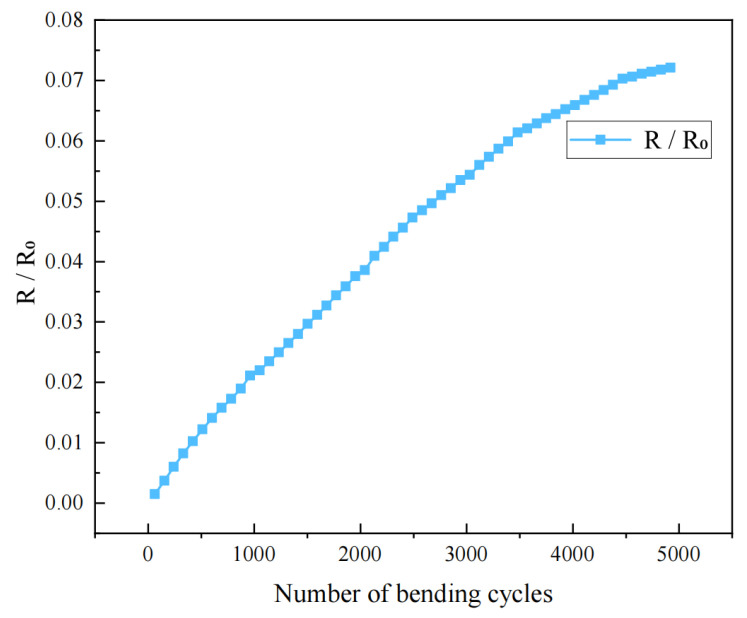
Repeated bending cycle performance of ITO/Ag/ITO/PI multilayer film.

The bending method is as shown in Figure 6. The bending durability of the thin-film sensor was preliminarily evaluated by monitoring the normalized resistance variation during cyclic bending. The results are shown in Figure 7.

As the bending cycle number increased from 0 to 5000, the normalized resistance gradually increased and then tended to level off at higher cycle numbers, with a total variation of approximately 0.07. This result indicates that the sensor maintained electrical continuity during repeated bending deformation, demonstrating a certain degree of mechanical flexibility. However, the gradual increase in resistance also suggests the existence of accumulated electrical drift, which may originate from microstructural evolution of the conductive pathway, interfacial degradation, or microcrack formation under cyclic strain. The cumulative electrical drift under repeated bending is likely associated with coupled conductive-path evolution, strain-induced microcrack accumulation, and interfacial degradation. A slight resistance decrease may occur at the initial stage because local deformation temporarily improves electrical contact, whereas continued loading progressively destabilizes the conductive network. As cracks nucleate and propagate, the conductive bridging effect of overlapped fractured segments is gradually lost. Meanwhile, surface defects facilitate oxygen/moisture ingress, weaken the ITO/Ag interfacial adhesion, promote Ag agglomeration, and induce local buckling or delamination. These processes collectively disrupt charge-transport continuity and eventually lead to irreversible resistance drift under repeated flexural loading [43,44,45]. Therefore, although the sensor shows acceptable bendability, its long-term mechanical reliability still requires further improvement.

## 5. Discussion

This study provides a prototype-oriented framework that combines a hybrid underground communication architecture with flexible wearable sensing modules for mine-rescue applications. Compared with conventional mine-rescue studies that mainly focus on communication support or environmental monitoring, the present work extends the design perspective toward rescuer-oriented physiological monitoring. The proposed Wi-Fi/optical-fiber architecture offers a practical combination of local deployment flexibility and backbone transmission reliability, while the GPANI/PVB-based temperature sensor and the ITO/Ag/ITO-based optoelectronic module provide preliminary support for flexible wearable integration. In particular, the temperature sensor showed a clear temperature-dependent resistance response, and the optoelectronic module maintained acceptable electrical continuity under repeated bending, indicating the potential of the selected materials and structures for dynamic rescue conditions.

However, the current work should still be regarded as a study of architectural design and component-level validation rather than a fully verified rescue-monitoring system. Real-time end-to-end transmission of physiological data from the wearable node to the surface command center has not yet been demonstrated, and complete emitter–detector–readout validation against reference physiological instruments remains unavailable. In addition, the pressure-related influence on the temperature-sensitive layer and the resistance drift observed during cyclic bending indicate that further optimization of sensing selectivity and long-term reliability is still required. Although the present study confirms the basic temperature-response behavior of the sensor, its reproducibility and thermal stability under repeated thermal loading remain to be systematically evaluated. In future work, at least five heating–cooling cycles will be performed to assess cycle-to-cycle consistency, baseline drift, and signal stability, while comparison of the heating and cooling response curves will help identify possible hysteresis behavior. Future studies should therefore focus on complete hardware integration and simulated mine-rescue experiments under representative underground conditions, with particular attention to transmission stability, physiological signal integrity, agreement with reference devices, and long-duration reliability under repeated mechanical and thermal loading.

## 6. Conclusions

A prototype-oriented mine-rescue monitoring framework combining hybrid communication architecture and flexible sensing modules was presented in this study. A GPANI/PVB-based temperature sensor and an ITO/Ag/ITO-based PPG-oriented flexible optoelectronic module were developed and preliminarily validated. The temperature sensor showed a clear temperature-dependent resistance response, and the optoelectronic module maintained acceptable electrical continuity under repeated bending. These findings provide a component-level technical basis for future mine-rescue monitoring, while full system integration and end-to-end validation remain subjects of future work.

## Figures and Tables

**Table 1:** Common underground mine wired communication networks [14,15].

Technology Type	Key Advantages	Main Limitations	Typical Applications
Coaxial Cable	High bandwidth, strong anti-interference, long-distance transmission	Difficult wiring, high cost, heavy weight	TV signals, broadband access, LAN
Twisted Pair	Low cost, good anti-interference, easy installation	Short distance (<100 m), limited bandwidth	Telephone lines, office LAN, digital communication
Fiber Optic	Ultra-high bandwidth (Tbps-level), zero interference, high security	Complex installation, high cost, fragile	Internet backbone, mine rescue communications, long-distance transmission

**Table 2:** Comparison table of core parameters of four wireless communication technologies [16,17,18,19].

Type	Transmission Rate	Coverage Range	Networking Flexibility
Wi-Fi	Up to 54 Mbps [19] (IEEE Std 802.11g-2003)	Tens of meters (indoor)	Ad hoc/infrastructure modes; no cables
RFID	Low (LF); higher (HF)	LF: short range; HF: 1 m; UHF: several meters	Reader-tag required; no self-organization
Bluetooth	Up to 3 Mbps (classic)	Around 10 m	Point-to-point/star; needs central device
ZigBee	250 kbps (2.4 GHz)	10–100 m (extendable to 1–3 km)	Star/tree/mesh;strong self-organization (65 k nodes)

**Table 3:** Equipment details of mixed network communication system for mine rescue.

Device	Function
Mining Intrinsically Safe Router (Disaster Area)	Entering the incident area with the rescue team and maintaining real-time data transmission with the downhole base during the rescue.
Mining Intrinsically Safe Router (Base)	Placed at the underground rescue base to maintain real-time data transmission between the rescue team and the underground base
Mining Intrinsically Safe Mobile Phone	Entering the accident area with the rescue team, collecting audio/video data and other monitoring data during the rescue process, and intercom between team members for emergency communication.
Mining Intrinsically Safe Tablet PC	Placed in the underground rescue base, to maintain real-time audio and video communications between the base and the rescue team and other safety information real-time monitoring
Gas Detector	With the rescue team into the accident area, on the way and the accident point real-time collection of environmental data (including temperature, humidity, carbon monoxide, methane, hydrogen sulphide, and oxygen levels) and transmission of these data back to the base.
Oxygen Respirator	Monitor the oxygen supply status of the rescue team members and transmit the supply data back to the underground rescue base in real time.
Vital Signs Sensor	Real-time monitoring of the rescue team members’ vital signs, the team members’ vital signs data transmitted back to the underground rescue base

**Table 4:** Thermal indices [17,18,20,21,22,23].

Monitoring Parameter	Alert Level	Action Level	Withdrawal Level	Recommended On-Site Response
Core Temperature (T_*c*_)	≥37.5–37.8 °C	≥38.0 °C	≥38.5 °C (continuously rising)	Reduce workload, initiate active cooling, and hydrate
Estimated Sweat Rate	≥1.0 L/h	≥1.5 L/h	≥2.0 L/h or signs of dehydration	Forced hydration and electrolyte replacement
Subjective Thermal Strain (Borg RPE/Thermal Sensation)	Noticeable heat discomfort	Dizziness, reduced attention	Confusion or impaired coordination	Immediate withdrawal and observation

**Table 5:** Heart rate–based threshold criteria for physiological monitoring in mine rescue operations [24,25,26,27,28].

Classification Level	Indicator	Threshold	Physiological Interpretation
Level I: Moderate-to-High Workload	Relative heart rate ($HR_{\max}$)	≥$78\%\;HR_{\max}$	Represents the average operational intensity observed in mine rescue activities
Level II: High Workload	Relative heart rate ($HR_{\max}$)	≥$85\%\;HR_{\max}$	Approaches the range of elevated metabolic and thermal strain
Level III: Very High Workload	Relative heart rate ($HR_{\max}$)	≥90–95% $HR_{\max}$	Near physiological limit; commonly observed peak levels during emergency operations
Level IV: Heat-related illness risk threshold	Age-adjusted maximal heart rate	HR≥180−age	Recommended sustained upper HR limit (ACGIH-based criterion)
Level V: Early warning threshold	Relative increase from resting HR ($\Delta HR_{\mathrm{rest}}$)	$\Delta HR_{\mathrm{rest}}\geq 132.9\%$	Optimal cut-off for predicting heat-related illness risk (sensitivity 75.5%, specificity 85.0%)
Level VI: Field peak reference	Absolute heart rate	≈177±23 bpm	Mean maximal HR observed during 30 min live-fire suppression
Level VII: Abnormal recovery	Recovery heart rate	Remains markedly above resting level 1–2 min post-task	Indicates inadequate cardiovascular recovery and sustained heat load

**Table 6:** Blood oxygen and oxygen indicators.

Indicator	Recommended Threshold	Occupational Health Significance
Ambient oxygen concentration	≥19.5% (safety lower limit)	Values below this level are defined as an oxygen-deficient work environment
Ambient oxygen concentration	19.5–23.5% (acceptable range)	Safe oxygen range for rescue operations
SpO_2_	<93% (warning threshold)	Indicates work-related hypoxemia
SpO_2_	≤80% (severe hypoxia)	May be associated with cognitive impairment
Oxygen concentration <16%	Cognitive and coordination impairment may occur	May compromise safe operational performance

**Table 7:** Key HiPIMS deposition parameters of the ITO/Ag/ITO multilayer film.

Parameter	Value	Unit
Base pressure	$<\;\;6.7\times 10^{-4}$	Pa
Average power for ITO deposition	$1.4\times 10^{3}$	W
Average power for Ag deposition	$1.0\times 10^{3}$	W
Ar flow rate for Ag deposition	(3.33–$6.67)\times 10^{-7}$	m^3^ s^−1^
Ar flow rate for ITO deposition	(3.33–$5.00)\times 10^{-7}$	m^3^ s^−1^
O_2_ flow rate for ITO deposition	(3.33–$8.33)\times 10^{-9}$	m^3^ s^−1^
Substrate temperature	<65	°C
Deposition rate of ITO	$8.1\times 10^{-10}$	m s^−1^
Deposition rate of Ag	$2.1\times 10^{-9}$	m s^−1^

**Table 8:** Recommended design targets for the 660 nm and 940 nm LEDs in the dual-wavelength PPG/SpO_2_-oriented flexible optoelectronic module [40,41,42].

Parameter	660 nm LED (Red Channel)	940 nm LED (IR Channel)
Recommended wavelength	655–660 nm	940 nm
Half-angle	$\pm 60^{\circ }$	$\pm 60^{\circ }$
Typical radiant flux at 20 mA	∼16 mW	∼11 mW
Typical forward voltage at 20 mA	∼1.9 V	∼1.3 V
Recommended drive mode	Pulsed constant-current drive	
Recommended peak current window	20–40 mA	
Recommended pulse width	118–215 μs	
Recommended timing sequence	Red pulse → IR pulse → ambient/off sampling	
Recommended driver architecture	Constant-current LED driver + MCU timing control + synchronized analog front-end/readout	

**Table 9:** Electrical properties of the fabricated transparent conductive multilayers.

Sample	Thickness (nm)	Sheet Resistance (Ω/sq)	Resistivity (Ω·m)
ITO	200	7746	$1.6\times 10^{-3}$
ITO/Ag/ITO	20/10/20	23.7	$1.19\times 10^{-6}$
ITO/Ag/ITO	20/20/20	6.5	$3.90\times 10^{-7}$
ITO/Ag/ITO	30/10/30	23.7	$1.66\times 10^{-6}$
ITO/Ag/ITO	30/20/30	6.5	$5.20\times 10^{-7}$

## Data Availability

The data presented in this study are not publicly available due to commercial restrictions. The data that support the findings are available from the corresponding author, P.C., upon reasonable request.

## Abbreviations

The following abbreviations are used in this manuscript:

PPG	photoplethysmography
(CSMA/CA)	Carrier Sense Multiple Access with Collision Avoidance
OFDM	Orthogonal Frequency Division Multiplexing
HR	Heart Rate
